# Efemp1 and p27^Kip1^ modulate responsiveness of pancreatic cancer cells towards a dual PI3K/mTOR inhibitor in preclinical models

**DOI:** 10.18632/oncotarget.859

**Published:** 2013-02-26

**Authors:** Sandra Diersch, Wenzel Patrick, Melanie Szameitat, Philipp Eser, Mariel C. Paul, Barbara Seidler, Stefan Eser, Marlena Messer, Maximilian Reichert, Philipp Pagel, Irene Esposito, Roland M. Schmid, Dieter Saur, Günter Schneider

**Affiliations:** ^1^ II. Medizinische Klinik, Technische Universität München, München, Germany; ^2^ Gene Center Munich, Ludwig-Maximilians-Universität (LMU) München, München, Germany; ^3^ Division of Gastroenterology, Department of Medicine, Abramson Cancer Center, University of Pennsylvania, School of Medicine, Philadelphia, Pennsylvania, USA; ^4^ numares GmbH, Regensburg, Germany; ^5^ Institut für Allgemeine Pathologie und Pathologische Anatomie, Technische Universität München, München, Germany

**Keywords:** pancreatic cancer, PI3K, Efemp1, p27, Bez235

## Abstract

Pancreatic ductal adenocarcinoma (PDAC) remains a dismal disease with a poor prognosis and targeted therapies have failed in the clinic so far. Several evidences point to the phosphatidylinositol 3-kinase (PI3K)-mTOR pathway as a promising signaling node for targeted therapeutic intervention. Markers, which predict responsiveness of PDAC cells towards PI3K inhibitors are unknown. However, such markers are needed and critical to better stratify patients in clinical trials.

We used a large murine Kras^G12D-^ and PI3K (p110α^H1047R^)-driven PDAC cell line platform to unbiased define modulators of responsiveness towards the dual PI3K-mTOR inhibitor Bez235. In contrast to other tumor models, we show that Kras^G12D-^ and PI3K (p110α^H1047R^)-driven PDAC cell lines are equally sensitive towards Bez235. In an unbiased approach we found that the extracellular matrix protein Efemp1 controls sensitivity of murine PDAC cells towards Bez235. We show that Efemp1 expression is connected to the cyclin-dependent kinase inhibitor p27^Kip1^. In a murine Kras^G12D-^ driven PDAC model, p27^Kip1^ haploinsufficiency accelerates cancer development in vivo. Furthermore, p27^Kip1^ controls Bez235 sensitivity in a gene dose-dependent fashion in murine PDAC cells and lowering of p27^Kip1^ decreases Bez235 responsiveness in murine PDAC models.

Together, we define the Efemp1-p27^Kip1^ axis as a potential marker module of PDAC cell sensitivity towards dual PI3K-mTOR inhibitors, which might help to better stratify patients in clinical trials.

## INTRODUCTION

Pancreatic ductal adenocarcinoma (PDAC) remains a dismal disease with a median survival under six months and a five-year survival rate of 5% [1]. No effective therapies for locally advanced or metastatic tumors exist, demonstrating the need to define novel therapeutic strategies and markers, which predict responsiveness.

Phosphatidylinositol 3-kinases (PI3K) build a conserved group of lipid kinases, composed of catalytically and regulatory subunits, which phosphorylate the 3` hydroxyl group of phosphatidylinositols, whereby generating phophatidylinositol (3,4,5) trisphosphate (PI(3,4,5)P3) from PI(4,5)P2 at the inner plasma membrane [2]. Intracellular PIP3 levels are tightly regulated by the tumor suppressor phosphatase and tensin homolog deleted on chromosome 10 (PTEN), which dephosphorylates PIP3 and therefore terminates PI3K signaling [3]. PIP3-induced proximity between the phosphoinositide-dependent protein kinase 1 (PDK1) and the AKT kinase allows PDK1 to phophorylate threonine 308 of AKT1 [2]. The PTEN-PI3K-AKT-mTOR pathway is frequently deregulated in PDAC. At the genetic level, mutations in the catalytical active class Ia PI3K subunit p110α [4] (www.sanger.ac.uk/genetics/CGP/cosmic/) and the regulatory subunit p85α [5] as well as amplification of AKT2 [6] were described. Accordingly, mutant p110α^H1047R^ can initiate and drive the carcinogenesis in the murine pancreas and the PI3K-PDK1 pathway is an essential node for Kras^G12D^-driven murine PDAC [7]. Furthermore, PTEN expression is lost or significantly reduced in human PDAC cell lines and tumor specimens [8] and surrogate markers of PI3K activity, like phosphorylation of AKT [7, 9, 10], were commonly detected in human and murine PDAC. Of note, AKT phosphorylation is inversely correlated with survival of PDAC patients [9, 11, 12]. Functionally, PI3K signaling is linked to processes like proliferation, therapeutic/apoptosis resistance, control of metabolism and angiogenesis in PDAC [13].

Due to the frequent activation of the PI3K pathway, PI3K inhibitors are promising therapeutic targets in solid tumors [14] including PDAC [13]. In agreement, the imidazoquinoline dual PI3K-mTOR inhibitor Bez235 revealed efficacy in *in vivo* models of PDAC [15, 16]. Furthermore, the pan-class I PI3K inhibitor GDC 0941 prevented tumor progression in an endogenous genetically defined mouse model and a humanized primary orthotopic xenotransplant model of PDAC [7]. Nevertheless, markers, which predict and modulate the response towards PI3K-mTOR inhibitors in PDAC are ill defined. In an attempt to unbiased define modulators of PI3K inhibitor sensitivity, we used a large murine PDAC cell line platform. We demonstrate here that Efemp1 as well as p27^Kip1^ axis controls responsiveness of PDAC cells towards Bez235.

## RESULTS

### Murine PDAC cells are sensitive to the dual PI3K/mTor Inhibitor Bez235

**Figure 1:**
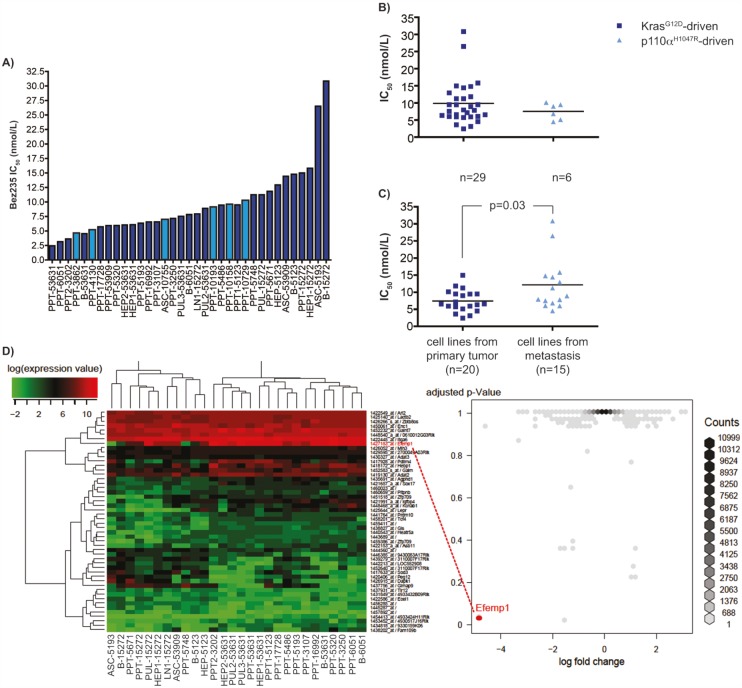
Bez235 IC_50_ values and differential expressed genes A) IC_50_ values of murine PDAC cell lines. 29 murine Kras^G12D^-driven (dark blue) or 6 p110α^H1047R^-driven (light blue) PDAC cell lines were treated with different concentrations of Bez235 and viability was determined after 72 hours using MTT assay`s. IC_50_ values were calculated using a non-linear regression analysis. B) Comparison of the IC_50_ values of Kras^G12D^-driven (dark blue) (n=29) or p110α^H1047R^-driven (light blue) (n=6) PDAC cell lines. Shown is the mean IC_50_ value of both groups. The p value is indicated. C) Comparison of the IC_50_ values of cell lines derived from primary PDAC (dark blue) (n=20) or metastasis (light blue) (n=15). Shown is the mean IC_50_ of both groups. The p value is indicated. D) Differential expression analysis of transcriptome profiles of 18 murine PDAC cell lines with a Bez235 IC_50_ < 10 nM and 10 murine PDAC cell lines with a Bez235 IC_50_ > 10 nM. Volcano-plot (right) showing the fold-change and p-value for all probesets calculated by comparing the two groups of samples. Most probesets (dark-grey) show no change in expression levels but some show observable higher probability to be differentially expressed. Efemp1 (log fold-change -4.7, p-value 0.02) stands out from the rest showing high expression levels in Bez235 sensitive murine PDAC cells and low expression in Bez235 insensitive murine PDAC cells. The 50 probesets with the highest fold-change are shown in a heatmap (left).

To determine the sensitivity of murine Kras^G12D^-driven or p110α^H1047R^-driven PDAC cells towards the dual PI3K/mTor inhibitor Bez235, we treated 35 cell lines with Bez235 for 72 hours. Viability was measured using MTT assays and the IC_50_ values were calculated using a non-linear regression analysis [17]. IC_50_ values between 2.4 nmol/L for the most sensitive up to 30.8 nmol/L were determined (figure 1A). Statistics can be found in supplemental table 1. No statistically significant difference in the mean IC_50_ values of murine Kras^G12D^-driven (mean IC_50_ value: 9.85 +/− 1.15 nmol/L) and p110α^H1047R^-driven (mean IC_50_ value: 7.51 +/− 0.97 nmol/L) PDAC cells was detected (figure 1B), arguing that the PI3K pathway is equally important to maintain viability in both models investigated. Interestingly, cell lines isolated from metastases reveal significantly higher IC_50_ values (mean IC_50_ value: 12.15 +/− 1.97 nmol/l) compared to cell lines isolated from primary PDAC (mean IC_50_ value: 7.43 +/− 0.72 nmol/L) (figure 1C). In contrast to the high sensitivity of the murine PDAC cell lines towards Bez235, IC_50_ values for the mTOR inhibitor Rad001 are high ranging from 0.28 to 6.49 μmol/L (supplemental table 1), which might argue for a significant contribution of the PI3K inhibition for the Bez235 sensitivity.

To unbiased find genes differentially expressed in murine PDAC cells sensitive to Bez235, we used microarrays available from 28 murine PDAC cell lines. We defined two groups according to an Bez235 IC_50_ cutoff of 10 nmol/L that best separates the available 28 cell lines with high and low Bez235 IC_50_ values. The 50 most significant genes that are differentially expressed in cell lines with low and high Bez235 IC_50_ values are shown in figure 1D. The gene, which was statistically significantly differentially expressed between cells with a low and high IC_50_ value and revealed the greatest expression difference in both groups, was the EGF-containing fibulin-like extracellular matrix protein 1 (Efemp1/Fibulin3) gene (log fold-change -4.7, p-value 0.02) (figure 1D).

### Efemp1 expression correlates with Bez235 IC50 values

**Figure 2:**
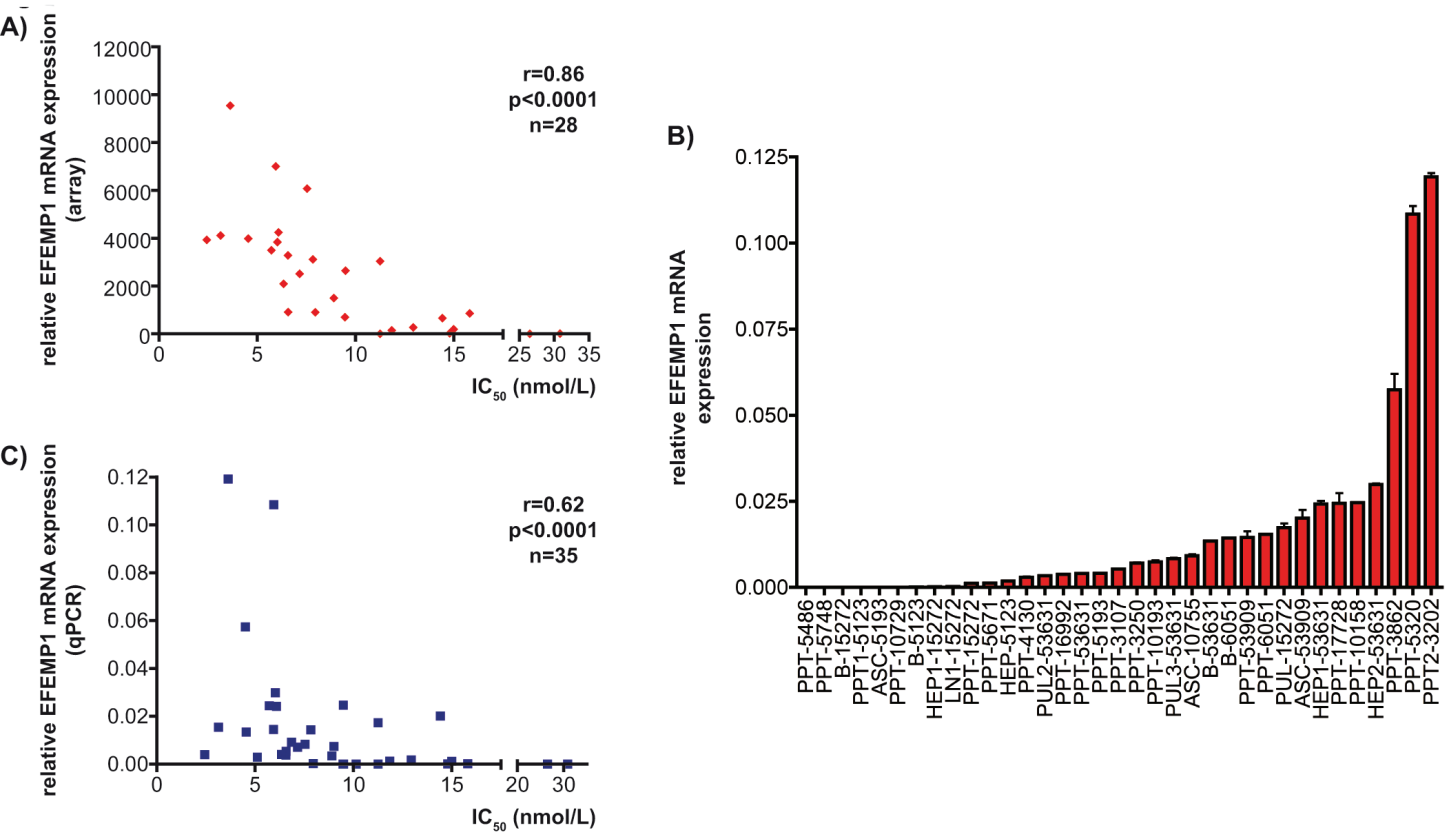
Efemp1 expression correlates with Bez235 IC_50_ values A) Bez235 IC_50_ values of 28 murine PDAC cell lines were correlated with relative Efemp1 expression from the microarray dataset. Spearman r and statistics are indicated. B) Efemp1 mRNA expression of 35 murine PDAC cell lines was determined by qPCR using cyclophilin A mRNA as reference. C) Bez235 IC_50_ values of 35 murine PDAC cell lines were correlated with relative Efemp1 expression determined by qPCR. Spearman r and statistics are indicated.

Efemp1 belongs to the Fibulin protein family of secreted glycoproteins, which are components of the extracellular matrix [18]. When array mRNA expression data of 28 cell lines for Efemp1 were correlated with Bez235 IC_50_ values, a Spearman correlation coefficient of r=0.86 (p<0.0001) was calculated (figure 2A). To corroborate the array expression data, we quantified Efemp1 mRNA expression in 35 murine PDAC cell lines using qPCR (figure 2B). Again, a significant correlation of Efemp1 with Bez235 IC_50_ values was evident (Spearman correlation coefficient of r=0.62; p<0.0001) (figure 2C), arguing that Efemp1 is a marker for Bez235 sensitivity.

### Efemp1 increases sensitivity towards Bez235

**Figure 3:**
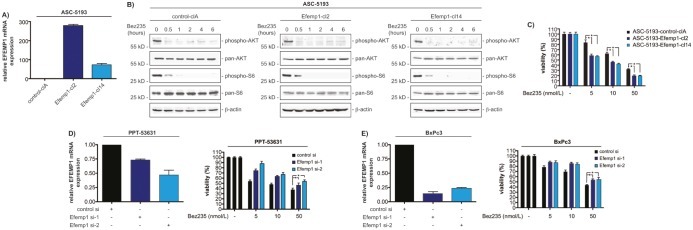
Efemp1 expression modulates Bez235 responsiveness A) ASC-5193 cells were stably transfected with a control (control-clA) or an Efemp1 expression vector (Efemp1-cl2 and Efemp1-cl14). Efemp1 mRNA expression was determined by qPCR using cyclophilin A mRNA as reference. B) ASC-5193 control-clA, Efemp1-cl2 and Efemp1-cl14 cells were treated with 50 nmol/L Bez235 over time as indicated. Western blots determine phosphorylation of AKT (S473) and S6 (S235/236) as well as expression of pan-AKT and pan-S6. β-actin controls equal protein loading. C) ASC-5193 control-clA, Efemp1-cl2 and Efemp1-cl14 were treated with Bez235 for 72 hours as indicated. Afterwards viability was measured using MTT assays. D) *Left graph*: PPT-53631 cells were transfected with control siRNA or two specific Efemp1 siRNAs. After 72 hours relative Efemp1 mRNA expression was determined by qPCR using cyclophilin A mRNA as reference. *Right graph*: PPT-53631 cells were transfected with control siRNA or two specific Efemp1 siRNAs. After 24 hours cells were treated for 72 hours as indicated. Afterwards viability was measured using MTT assays. E) *Left graph*: BxPc3 cells were transfected with control siRNA or two specific Efemp1 siRNAs. After 72 hours relative Efemp1 mRNA expression was determined by qPCR using cyclophilin A mRNA as reference. *Right graph*: BxPc3 cells were transfected with control siRNA or two specific Efemp1 siRNAs. After 24 hours cells were treated for 72 hours as indicated. Afterwards viability was measured using MTT assays.

To demonstrate a functional relevance of Efemp1 modulating Bez235 sensitivity we used gain- and loss-of-function studies. We stably transfected the murine ASC-5193 cell line (IC_50_ high) with an Efemp1 expression vector. Figure 3A, demonstrates increased expression of the Efemp1 mRNA in two clones compared to control transfected cells. We were not able to detect murine Efemp1 protein with the available Efemp1 antibodies. Bez235 leads to PI3K pathway inhibition, as measured by dephosphorylation of AKT and S6, irrespectively of the stable transfection of Efemp1 (figure 3B). However, as suggested by the correlation of Efemp1 expression with Bez235 IC_50_ values, elevation of Efemp1 expression increased the sensitivity of murine ASC-5193 towards Bez235 (figure 3C).

In addition to the gain-of-function approach, we used RNAi to demonstrate the effects of Efemp1 towards the Bez235 sensitivity in the murine PPT-53631 cells (IC_50_ low) as well as in human BxPc3 cells. Knockdown of Efemp1 in PPT-53631 and BxPc3 cells was demonstrated at the level of mRNA using qPCR in PPT-53631 (figure 3D, left graph) and BxPc3 cells (figure 3E, left graph). Reducing Efemp1 expression decreased Bez235 sensitivity in PPT-53631 (figure 3D, right graph) and BxPc3 cells (figure 3E, right graph). Together, these data demonstrate that Efemp1 modulates sensitivity towards Bez235 in cell-based PDAC models.

### Efemp1 increases the expression of the cyclin-dependent kinase inhibitor p27^Kip1^

**Figure 4:**
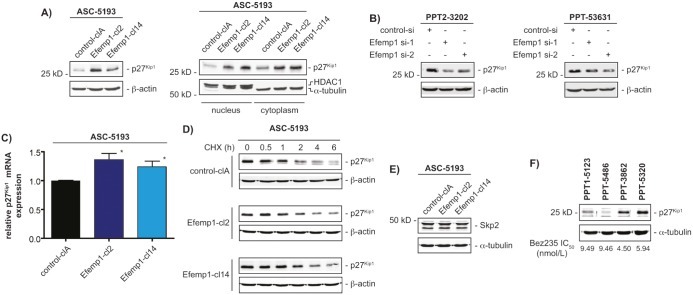
Efemp1 controls expression of p27^Kip1^ A) *Left panel*: Western blot detected expression of p27^Kip1^ in whole cell extracts of ASC-5193 cells stable transfected with a control (control-clA) or an Efemp1 expression vector (Efemp1-cl2 and Efemp1-cl14). β-actin controls equal protein loading. *Right panel*: Western blot of p27^Kip1^ expression in cytoplasmatic and nuclear extracts of ASC-5193 cells transfected with a control (control-clA) or an Efemp1 expression vector (Efemp1-cl2 and Efemp1-cl14). HDAC1 and α-tubulin controls cytoplasmatic and nuclear fractionation and loading. B) PPT2-3202 and PPT-53631 cells were transfected with a control siRNA or two specific Efemp1 siRNAs. After 48 hours western blot detected expression of p27^Kip1^. β-actin controls equal protein loading. C) ASC-5193 cells were stable transfected with a control (control-clA) or an Efemp1 expression vector (Efemp1-cl2 and Efemp1-cl14). p27^Kip1^ mRNA expression was determined by qPCR using cyclophilin A mRNA as reference. D) ASC-5193 control-clA, Efemp1-cl2 and Efemp1-cl14 cells were treated with cycloheximide (50 μg/ml) over time as indicated. Western blot detected expression of p27^Kip1^. β-actin controls equal protein loading. E) Skp2 western blot in ASC-5193 control-clA, Efemp1-cl2 and Efemp1-cl14 cells. β-actin controls equal protein loading. F) Western blot of p27^Kip1^ expression in murine PPT1-5123, PPT-5486, PPT-3862 and PPT-5320 PDAC cell lines. α-tubulin controls equal protein loading. Bez235 IC_50_ values for each cell line are depicted.

Since we have demonstrated that inhibition of the PI3K pathway in human PDAC models induces a cytostatic response with a G1-phase arrest in the cell cycle involving the cyclin-dependent kinase inhibitor p27^Kip1^ [19, 20] and p27^Kip1^ was recently shown to control sensitivity towards dual PI3K/mTOR inhibition [21], we investigated p27^Kip1^ in ASC-5193 overexpressing Efemp1. p27^Kip1^ protein levels were significantly elevated in stably Efemp1 transfected clones compared to the control clone (figure 4A). Increased p27^Kip1^ protein was distributed in the cytoplasm and the nucleus in the investigated ASC-5193 clones (figure 4A, right panel). Consistently, RNAi targeting Efemp1 leads to the downregulation of p27^Kip1^ protein expression in murine PPT-3202 and PPT-53631 cells (figure 4B). Knockdown in PPT2-3202 and PPT-53631 cells was controlled at the level of Efemp1 mRNA expression (data not shown).

To investigate how Efemp1 controls p27^Kip1^ protein expression, we measured p27^Kip1^ mRNA expression and protein turnover. A slight increase in p27^Kip1^ mRNA expression was observed in stably Efemp1 transfected ASC-5193 cells compared to controls (figure 4C). Protein turnover of p27^Kip1^ was not changed in ASC-5193 cells, irrespectively of Efemp1 expression (figure 4D). Consistently, the expression of a main regulator of p27^Kip1^ degradation in PDAC cells, the F-box protein S-phase kinase associated protein 2 (Skp2) [19], was not changed in the stable Efemp1 transfected ASC-5193 clones (figure 4E). To confirm the correlation of p27^Kip1^ and Bez235 responsiveness, we measured p27^Kip1^ protein expression in murine PDAC cell lines with different IC_50_ values. Consistent with the data from stable Efemp1 transfected ASC-5193 cells, p27^Kip1^ was higher expressed in murine PDAC cell lines with lower Bez235 IC_50_ values (figure 4F).

### p27^Kip1^ haploinsufficiency accelerates cancer development in a murine Kras^G12D^-driven PDAC model

**Figure 5:**
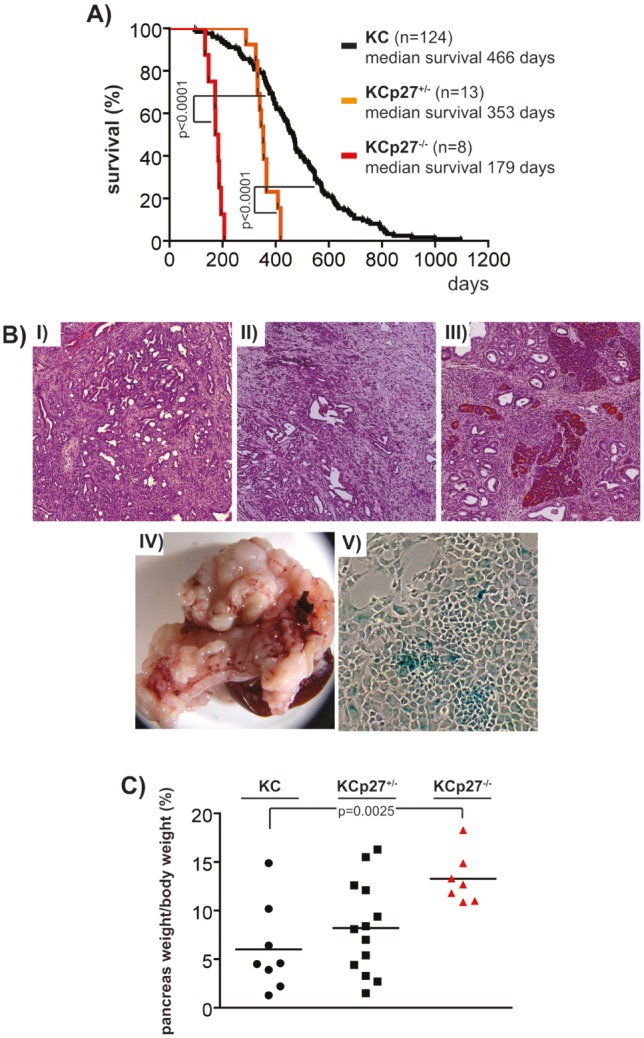
p27^Kip1^ haploinsufficiency accelerates Kras^G12D^-dependent cancer development in the murine pancreas A) Kaplan-Meier survival curves of the indicated genotypes. p values of the log-rank test are indicated. B) Microscopie of the PDAC of *I)* KC mice and *II)* KCp27^Kip1+/−^ mice and of the pancreas with typical pre-malignant changes observed in *III)* KCp27^Kip1+/−^ mice. (original magnification 50x) *IV)* Macroscopic appearance of the pancreas of KCp27^Kip1−/−^ mice. *V)* Microscopic images of epithelial cells isolated from KCp27^Kip1−/−^ mice in culture stained for SA-β-galactosidase. (original magnification 100x). C) Pancreas weight/body weight % of the indicated genotypes. p value of the Student`s t-test is indicated.

To demonstrate the modulatory effect of p27^Kip1^ towards the PI3K inhibitor response at the genetic level, we attempted to generate p27^Kip1+/−^ and p27^Kip1−/−^ murine PDAC cell lines. We crossed the *p27^Kip1^* knockout mouse line [22] into the *Ptf1a^Cre/+^;LSL-Kras^G12D/+^* (KC mice thereafter) mouse model of PDAC [23]. Kaplan-Meier analysis demonstrated that p27^Kip1^ haploinsufficiency accelerates cancer development in the investigated model (figure 5A). PDACs developing in KCp27^Kip1+/−^ (figure 5B, II) mice are differentiated ductal adenocarcinomas as observed in KC mice (figure 5B, I). Furthermore, there is a tendency of an increased macroscopic metastasis rate into lymph nodes, liver and lungs in KCp27^Kip1+/−^ mice (KC, n=43, 39.1% macroscopic metastasis; KCp27^Kip1+/−^, n=13, 69.2% macroscopic metastasis; Fisher`s exact test p=0.067). Although KCp27^Kip1−/−^ mice die with a median survival of 179 days (figure 5A), no invasive cancers were detected (figure 5B, III). Histological, pancreata of KCp27^Kip1−/−^ mice show desmoplastic reaction with numerous PanIN lesions (figure 5B, III). Pancreata of KCp27^Kip1−/−^ mice are massively enlarged (figure 5B, IV and 5C), leading to obstruction of the surrounding GI-tract and subsequent death. Furthermore, cell lines isolated from KCp27^Kip1−/−^ tumors undergo a senescence crisis *in vitro* (figure 5B, V).

### p27^Kip1^ expression levels determine Bez235 sensitivity

**Figure 6:**
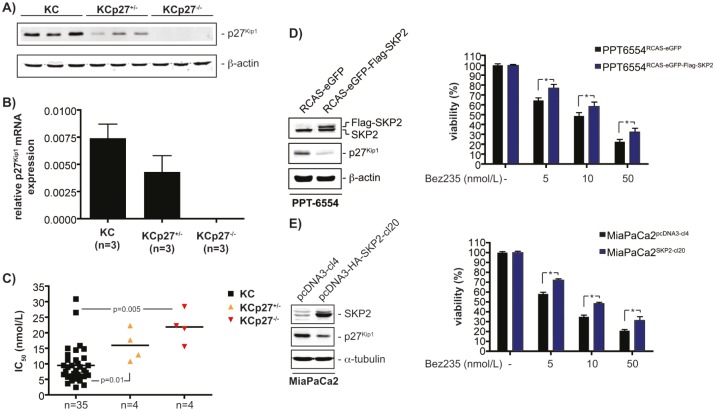
p27^Kip1^ controls responsiveness of PDAC cells towards Bez235 A) Western blot detects expression of p27^Kip1^ in the murine cell lines with the indicated genotype. β-actin controls equal protein loading. B) p27^Kip1^ mRNA expression in cell lines with the indicated genotype was determined by qPCR using cyclophilin A mRNA as reference. C) IC_50_ values of four KCp27^Kip1+/−^ and four KCp27^Kip1−/−^ cell lines were determined and compared to the IC_50_ values of 35 murine PDAC cell lines. The p value is indicated. D) *Left panel*: Murine PPT-6554 cells were transduced with RCAS-Flag-Skp2-EGFP virus or the RCAS-EGFP control retrovirus. Western blot detected expression of Skp2 and p27^Kip1^. β-actin controls equal protein loading. *Right graph*: Murine PPT-6554 cells transduced with RCAS-Flag-Skp2-EGFP virus or the RCAS-EGFP control retrovirus were treated with Bez235 as indicated or were left as vehicle treated controls. After 72 hours viability was determined with MTT assays. E) *Left panel*: Western blot of Skp2 and p27^Kip1^ expression in MiaPaCa2 cells stably transfected with a control (pcDNA3-cl4) or a HA-tagged Skp2 expression vector (pcDNA3-HA-Skp2-cl20). α-tubulin controls equal protein loading. *Right graph*: MiaPaCa2 cells stably transfected with a control (pcDNA3-cl4) or a HA-tagged Skp2 expression vector (pcDNA3-HA-Skp2-cl20) were treated with Bez235 as indicated or were left as vehicle treated controls. After 72 hours viability was determined with MTT assays.

Expression levels of p27^Kip1^ were downregulated at protein (figure 6A) and mRNA (figure 6B) level in cell lines from KCp27^Kip1+/−^ mice compared to KC cell lines. p27^Kip1^ protein and mRNA expression was absent in cell lines from KCp27^Kip1−/−^ mice (figure 6A and 6B). To prove the contribution of p27^Kip1^ towards the control of Bez235 sensitivity, IC_50_ values of four cell lines from KCp27^Kip1+/−^ as well as cell lines from KCp27^Kip1−/−^ tumors were determined and compared to the IC_50_ values of p27^Kip1^-proficient PDAC cell lines (n=35). As shown in figure 6C, lowering p27^Kip1^ expression increased IC_50_ values in a gene dose-dependent manner.

To further establish the role of p27^Kip1^ as a regulator of Bez235 sensitivity, we reduced the expression of p27^Kip1^ by increasing Skp2 expression in the murine PPT-6554 cell line and the human MiaPaCa2 cell line. Increasing the expression of Skp2 leads to a significant downregulation of p27^Kip1^ in PPT-6554 (figure 6D, left panel) and MiaPaCa2 cells (figure 6E, left panel). Lowering p27^Kip1^ by Skp2 decreases sensitivity of PPT-6554 (figure 6D, right graph) and MiaPaCa2 (figure 6E, right graph) cells for Bez235, further supporting the notion that p27^Kip1^ is an important regulator of Bez235 sensitivity in cell-based PDAC models.

## DISCUSSION

A goal of personalized medicine is to stratify patients to maximize therapeutic responses to specific targeted therapies. Therefore, there is the need to gain insights into the molecular mode of action of targeted therapies and to define markers and modulators of responsiveness. Here we used a large murine PDAC cell line platform to unbiased find modulators of the response towards the dual PI3K/mTOR inhibitor Bez235. By the use of gain- and loss-of-function experiments, we demonstrate that Efemp1 and p27^Kip1^ are involved in the control of Bez235 sensitivity of PDAC cells in this preclinical model.

In several tumor entities predictive markers for responsiveness towards PI3K-AKT pathway inhibition were described, whereat genetic lesions in the components of the PI3K-AKT pathway were found positively correlated with PI3K inhibitor sensitivity. Consistently, p110α mutation and/or loss of PTEN expression may characterize sensitive cancer cells [24-29]. Although a low number of patients were included, a recent clinical trial reported increased response rates towards PI3K-AKT-mTOR inhibitors, when patients with gynecologic malignancies were stratified with regard to the presence of a p110α mutation [30]. Consistent, an increased response rate towards PI3K-AKT-mTOR inhibitors in p110α mutated cancers was recently confirmed in a retrospective study [31]. In addition to genetic changes, surrogate marker of PI3K pathway activation, like phosphorylation of AKT, denote increased flux through the signaling pathway and therefore may indicate PI3K inhibitor sensitivity in some cancer models investigated [32, 33]. However, discrepant results for genetic markers as well as pathway activation markers to predict PI3K inhibitor sensitivity were reported. For example, no correlation of the PTEN status with the responsiveness of breast cancer cells towards GDC 0941 was detected [26] and neither p110 nor PTEN mutations were correlated with PI3K inhibitor sensitivity in the JFCR39 human cancer cell line panel [33]. Furthermore, no correlation between basal activity of PI3K-AKT signaling and the potency of Bez235 to induce dephosphorylation of AKT was observed [24] and the extend of inhibition of the PI3K/AKT signaling pathway at the biochemical level induced by PI3K inhibitors does not predict the biological response [34]. These results argue that tumor-type specificities contribute to the discrepant results with respect to markers predicting the response towards PI3K inhibitors. This notion is now also corroborated by our results. Concordantly, mutations in the oncogenes KRAS or BRAF are reported to confer resistance towards PI3K pathway inhibitors [24, 26, 33, 35, 36]. Consistent, murine p110α^H1047R^-driven lung cancers respond to Bez235 therapy, whereas murine Kras^G12D^-driven lung cancers do not [37]. In contrast our preclinical murine PDAC cell line platform demonstrates that murine PDAC cells are equally sensitive towards Bez235, irrespectively whether they are initiated and driven by p110α^H1047R^ or Kras^G12D^ oncogenes. These data argue that I) the PI3K pathway is an important node and target for therapeutic intervention in PDAC and II) that each tissue has its unique and specific signaling requirements for tumor maintenance. Furthermore, tumor entity specific markers might be necessary to predict sensitivity towards PI3K inhibition.

The combined partial/complete response rate of p110α mutated cancers towards PI3K-AKT-mTOR inhibitors is only 18% to 30% [30, 31], arguing that further markers predicting responsiveness towards these targeted therapies are needed. We detected the Efemp1 gene differentially expressed in murine PDAC cells with low and high sensitivity for Bez235. Efemp1 belongs to the fibulin protein family, glycoproteins of the extracellular matrix consisting out of Fibulin 1 to 7 [18]. Efemp1 can conduct pro-tumorigenic as well as tumor-suppressive functions dependent on the cancer type. In PDAC, Efemp1 mRNA was upregulated in 13 of 15 investigated cancer specimens [38]. Efemp1 mRNA expression was variable increased ranging from 1.5 to over 10 fold induction in cancers compared to normal tissues [38]. This is consistent with our expression analysis in the murine PDAC cell line platform, which also demonstrates a variable expression of the Efemp1 mRNA. The variable expression of Efemp1 might argue that high Efemp1 expression characterizes a subtype of PDAC. Since our gain- and loss-of-function studies shows that high Efemp1 expression increases the responsiveness towards Bez235, this PDAC subtype might benefit from PI3K inhibitors.

Considering recent observations that Efemp1 can easily be detected in serum of patients with pleural mesothelioma and that high serum Efemp1 levels discriminates asbestos-exposed persons without mesothelioma from asbestos-exposed persons with mesothelioma [39], points to an avenue to include Efemp1 serum levels as a biomarker in trials with PI3K-mTOR inhibitors in PDAC. However, high Efemp1 serum levels were not detected in breast cancer, prostate cancer, ovarian cancer, lung cancer (with effusion) or glioblastoma [39]. Therefore, the definite prove that a PDAC subgroup with high Efemp1 serum levels exists and responds to PI3K-mTOR inhibitors, awaits further clinical investigations.

Mechanistically, Efemp1 was shown to promote metastasis, cell cycle progression and apoptosis resistance in a human PDAC model [38]. To induce changes in cellular behavior of tumor cells, Efemp1 acts in a para- and autocrine fashion by modulating signaling pathways like the EGF receptor pathway [40] or the NOTCH pathway [41]. We detected that the cyclin-dependent kinase inhibitor p27^Kip1^ is connected to Efemp1 expression. Loss of p27^Kip1^ expression, which occurs in 50-70% of PDACs, is a marker for poor prognosis of the disease [42-44]. Consistent, we observed acceleration of the Kras^G12D^-driven carcinogenesis in the pancreas of p27^Kip1+/−^ mice. Furthermore, we provide genetic evidence that higher p27^Kip1^ expression increases the sensitivity of PDAC cells for Bez235, which might characterize p27^Kip1^ as a further predictive marker for PI3K-mTOR inhibitor sensitivity in PDAC. Interestingly, p27^Kip1^ was shown to be predictive for dual PI3K-mTOR inhibitor sensitivity in a cell-based model of pituitary adenomas from rats with multiple endocrine neoplasia-like syndrom [21] and p27^Kip1^ expression correlated with rapalog sensitivity in the NCI-60 cell line platform [45].

Whereas increasing Efemp1 expression induces p27^Kip1^ expression, and vice versa, reducing Efemp1 expression reduces p27^Kip1^ expression in the investigated murine cell lines, it is currently unknown whether p27^Kip1^ is the sole effector directed by Efemp1 to modulate PI3K-mTOR inhibitor sensitivity. Since the aim of the current study was to unbiased define markers for PI3K-mTOR inhibitor sensitivity in PDAC models, clarification of this topic awaits further experiments beyond the scope of the current manuscript.

Although we provide some evidence that Efemp1 and p27^Kip1^ are also relevant in human PDAC models to control the sensitivity towards the dual PI3K-mTOR inhibitor, sufficient validation of both markers depends on the availability of a large low-passaged human PDAC cell line platform currently unavailable in our laboratory. Furthermore, additional *in vivo* studies, beyond the scope of the current manuscript, are needed to additional define the predictive value of Efemp1 and p27^Kip1^ for PI3K-mTOR inhibitor sensitivity in pre-clinical settings.

In conclusion, we have defined the Efemp1-p27^Kip1^ axis as predictive for the sensitivity of PDAC cells for the dual PI3K-mTOR inhibitor Bez235 in a murine pre-clinical model. These observations might help to better stratify clinical trials with PI3K-mTOR inhibitors in PDAC.

## MATERIAL AND METHODS

### Compounds

Are described in supplementary methods.

### Cell lines, generation and culture of murine PDAC cells

Primary dispersed murine pancreatic cancer cells were established from genetically engineered Kras^G12D^- or p110α^H1047R^ -based mouse models of PDAC and cultivated as described [46, 47]. A description of the used murine cell lines can be found in supplemental table 2. Human PDAC cells (MiaPaCa2 and BxPc3) were cultured as described [46].

### Mouse strains

Mouse strains are described in supplementary methods. The strains were interbred to obtain mice with the respective genotypes as described [48]. All animal studies were conducted in compliance with European guidelines for the care and use of laboratory animals and were approved by the local authorities.

### Histochemistry and SA-β-galactosidase staining

For histopathological analysis, murine specimens were fixed in 4% formaldehyde, embedded in paraffin and sectioned (3 μm thick). Tumors were stained with haematoxylin and eosin as described [48, 49]. SA-β-galactosidase staining is described in supplementary methods.

### Total cell lysates, Nuclear extracts, Western blot and Viability assays

Whole cell lysates and nuclear extracts were prepared and western blots were carried out as recently described [46, 47, 50, 51]. Used antibodies are described in supplementary methods. Western blots were performed using Odyssey Infrared Imaging System (LI-COR Biosciences) as described [47]. Viability of the cells was measured using MTT-assays as described [46].

### RNA interference

siRNAs were transfected with polyethylenimine (Sigma-Aldrich) at a final concentration of 50 nM as described [52]. siRNAs were purchased from Eurofins (Ebersberg, Germany) and sequences are depicted in supplementary methods.

### Quantitative Reverse-Transcriptase PCR

Total RNA was isolated from pancreatic carcinoma cell lines using the RNeasy kit (Qiagen) following the manufacturer`s instructions. Quantitative mRNA analyses were performed as previously described using real-time PCR analysis (TaqMan, PE StepOnePlus, Real time PCR System, Applied Biosystems) [19]. Primers are depicted in supplementary methods.

### Gene Expression profiling and bioinformatics

Total RNA from murine PDAC cell lines was prepared using the RNeasy kit (Qiagen). Labeled cRNA was synthesized, hybridized onto GeneChip Mouse Genome 430 2.0 arrays (Affymetrix) [17]. Microarray analysis is described in supplementary methods. Microarray data were submitted to the GEO repository (Accession: GSE40609).

### Stable transfection, RCAS virus construction and RCAS virus transduction

Are described in supplementary methods.

### Statistical methods

A two-tailed Student`s t-test was used to test statistical significance. * denotes a p-value of at least <0.05. p-values were calculated with GraphPad Prism4 software. IC_50_ values were calculated with GraphPad Prism4 using a non-linear regression model. Kaplan-Meier survival curves were compared by log-rank test. All data were obtained from three independent experiments performed in triplicate, and the results are presented as mean and standard error of the mean (S.E.M).

## Supplementary Tables and Methods




