# Supplementary material for: Efemp1 and p27Kip1 modulate responsiveness of pancreatic cancer cells towards a dual PI3K/mTOR inhibitor in preclinical models
Source: Oncotarget. 2013 Feb 26;4(2):277–88. doi: 10.18632/oncotarget.859 (PMC3712573; doi:10.18632/oncotarget.859)
Supplement: Supplementary file 1 [file oncotarget-04-277-s001.doc]

Efemp1 and p27Kip1 modulate responsiveness of pancreatic cancer cells towards a dual PI3K/mTOR inhibitor in preclinical models – Diersch et al

Supplementary Table 1:

MTT BEZ235 Rad001

(72 h) IC_50_ (nmol/L) CI 95% IC_50_ (μmol/L) CI 95%

PPT-53631 2.43 1.65-3.57 0.54 0.30-0.98

PPT-6051 3.14 1.96-5.02 0.73 0.38-1.37

PPT2-3202 3.63 2.98-4.42 0.28 0.13-0.58

PPT-3862 4.50 3.26-6.22 n.d.

B-53631 4.54 2.99-6.90 0.29 0.14-0.59

PPT-4130 5.11 4.13-6.34 n.d.

PPT-17728 5.72 4.11-7.96 0.52 0.26-1.07

PPT-53909 5.93 4.63-7.60 1.26 0.77-2.07

PPT-5320 5.94 4.96-7.12 0.51 0.30-0.80

HEP2-53631 6.03 3.87-9.40 3.06 2.16-4.33

HEP1-53631 6.08 3.71-9.99 0.57 0.31-1.07

PPT-5193 6.34 3.95-10.19 0.97 0.63-1.48

PPT-16992 6.56 4.97-8.68 1.09 0.78-1.50

PPT-3107 6.57 4.18-10.31 0.53 0.32-0.87

ASC-10755 6.85 5.26-8.93 n.d.

PPT-3250 7.16 4.73-10.85 0.57 0.32-1.03

PUL3-53631 7.54 5.23-10.88 0.49 0.25-0.97

B-6051 7.83 5.35-11.46 0.72 0.46-1.13

LN1-15272 7.95 5.20-12.15 1.34 0.73-2.45

PUL2-53631 8.90 7.13-11.11 0.59 0.40-0.86

PPT-10193 9.00 6.47-12.51 n.d.

PPT-5486 9.46 7.26-12.31 1.28 1.08-1.51

PPT-10158 9.48 7.15-12.58 n.d.

PPT1-5123 9.49 7.56-11.90 1.37 1.02-1.84

PPT-10729 10.13 7.31-14.10 n.d.

PPT-5748 11.25 6.44-19.65 0.79 0.55-1.11

PUL-15272 11.25 7.92-15.97 1.01 0.72-1.43

PPT-5671 11.84 6.68-21.00 1.52 1.01-2.30

HEP-5123 12.94 9.80-17.08 1.17 0.66-2.08

ASC-53909 14.42 11.5-18.07 2.77 1.99-3.85

B-5123 14.79 9.30-23.52 1.25 0.81-1.93

PPT-15272 14.99 7.90-28.30 2.22 1.70-2.89

HEP1-15272 15.81 10.96-22.80 0.96 0.85-1.09

ASC-5193 26.51 19.96-35.23 3.46 1.94-6.15

B-15272 30.84 20.63-46.11 6.49 2.61-16.1

**Supplemental table 2**

**Cell Line Genotype**

PPT-3107 *Ptf1a^Cre/+^, LSL-Kras^G12D/+^*, *LSL-p53^R172H/+^*

PPT2-3202 *Ptf1a^Cre/+^, LSL-Kras^G12D/+^*, *LSL-PCNA^ATG-fLuc/+^*

PPT-3250 *Pdx1-Cre, LSL-Kras^G12D/+^*, *LSL-PCNA^ATG-fLuc/+^,*

*LSL-R26^Tva-lacZ/+^*

PPT-3862 *Ptf1a^Cre/+^, Pdx1-Cre, LSL-p110α^H1047R/+^*, *LSL-p53^R172H/+^*,

*LSL-R26^Tva-lacZ/+^*

PPT-4130 *Ptf1a^Cre/+^, LSL-p110α^H1047R/+^*, *LSL-p53^R172H/R172H^*

PPT1/B/HEP-5123 *Ptf1a^Cre/+^, LSL-Kras^G12D/+^*, *LSL-PCNA^IRES-fLuc/+^*

PPT/ASC-5193 *Ptf1a^Cre/+^, LSL-Kras^G12D/+^*, *LSL-p53^R172H/+^*

PPT-5320 *Ptf1a^Cre/+^, LSL-Kras^G12D/+^*, *LSL-PCNA^IRES-fLuc/+^*

PPT-5486 *Ptf1a^Cre/+^, LSL-Kras^G12D/+^*, *LSL-p53^R172H/+^*,

*LSL- PCNA^IRES-fLuc/+^*

PPT-5671 *Ptf1a^Cre/+^, LSL-Kras^G12D/+^*, *LSL-PCNA^ATG-fLuc/+^,* *LSL-R26^Tva-lacZ/+^*

PPT-5748 *Ptf1a^Cre/+^, LSL-Kras^G12D/+^*

PPT/B-6051 *Pdx1-Cre, LSL-Kras^G12D/+^*, *LSL-p53^R172H/+^*

PPT-6554 *Ptf1a^Cre/+^, LSL-Kras^G12D/+^*, *p53^lox/lox^*, *LSL-R26^Tva-lacZ/+^*

PPT-7662 *Ptf1a^Cre/+^, LSL-Kras^G12D/+^*, *LSL-R26^Tva-lacZ/+^*

PPT-8024 *Ptf1a^Cre/+^, LSL-Kras^G12D/+^*, *LSL-R26^Tva-lacZ/Tva-lacZ^*

PPT-8025 *Ptf1a^Cre/+^, LSL-Kras^G12D/+^*, *LSL-R26^Tva-lacZ/Tva-lacZ^*

PPT-10158 *Ptf1a^Cre/+^, LSL-p110α^H1047R/+^*, *LSL-R26^Tva-lacZ/+^*

PPT-10193 *Ptf1a^Cre/+^, Pdx1-Cre, LSL-p110α^H1047R/H1047R^*,

*LSL-p53^R172H/+^*

PPT-10729 *Ptf1a^Cre/+^, LSL-p110α^H1047R/H1047R^, LSL-p53^R172H/R172H^*

ASC-10755 *Ptf1a^Cre/+^, LSL-p110α^H1047R/H1047R^, LSL-p53^R172H/R172H^*

PPT/B/HEP1/PUL/LN1-15272 *Ptf1a^Cre/+^, LSL-Kras^G12D/+^*, *LSL-p53^R172H/+^*

PPT-16992 *Ptf1a^Cre/+^, LSL-Kras^G12D/+^*, *LSL-R26^Tva-lacZ/+^*

PPT-17728 *Ptf1a^Cre/+^, LSL-Kras^G12D/+^*, *LSL-R26^Tva-lacZ/+^*

PPT/B/HEP1/2/PUL2/3-53631 *Ptf1a^Cre/+^, LSL-Kras^G12D/+^*

PPT/ASC-53909 *Ptf1a^Cre/+^, LSL-Kras^G12D/+^*, *LSL-R26^Tva-lacZ/+^*

PPT-D146 *Ptf1a^Cre/+^, LSL-Kras^G12D/+^*, *p27^+/-^*

PPT/HEP1-D261 *Ptf1a^Cre/+^, LSL-Kras^G12D/+^*, *p27^+/-^*, *LSL-R26^Tva-lacZ/+^*

PPT1/PPT2-D341 *Ptf1a^Cre/+^, LSL-Kras^G12D/+^*, *p27^-/-^*

PPT-D380 *Ptf1a^Cre/+^, LSL-Kras^G12D/+^*, *p27^+/-^*, *LSL-R26^Tva-lacZ/+^*

PPT-D613 *Ptf1a^Cre/+^, LSL-Kras^G12D/+^*, *p27^-/-^*

PPT-D642 *Ptf1a^Cre/+^, LSL-Kras^G12D/+^*, *p27^-/-^*
