# Supplementary material for: Efemp1 and p27Kip1 modulate responsiveness of pancreatic cancer cells towards a dual PI3K/mTOR inhibitor in preclinical models
Source: Oncotarget. 2013 Feb 26;4(2):277–88. doi: 10.18632/oncotarget.859 (PMC3712573; doi:10.18632/oncotarget.859)
Supplement: Supplementary file 2 [file oncotarget-04-277-s002.pdf]

Efemp1 and p27Kip1 modulate responsiveness of pancreatic cancer cells towards a dual PI3K/mTOR inhibitor in preclinical models – Diersch et al

Supplementary Material and Methods

Compounds

The PI3K inhibitor Bez235 was kindly provided by Novartis (Novartis, Basel, Switzerland). Cycloheximide was purchased from Sigma-Aldrich (Sigma-Aldrich, Munich, Germany), Rad001 from LC Laboratories (Woburn, MA, USA) and 5-bromo-4-chloro-3-indolyl β-D-galactopyranoside (X-Gal) from peqLab (peqLab, Erlangen, Germany).

Mouse strains

LSL-Kras$^{G12D/+}$ [1], *Ptf1a*$^{Cre/+}$ [2], *Pdx1-Cre* [1], *LSL-p53*^{R172H} [3], LSL-R26$^{\text{Tva-lacZ}}$ [4], *LSL-p110a*^{H1047R} [5] and *p53*^{lox} [6] were described. p27$^{\text{Kip1}}$ knock-out mice were kindly provided by Dr. Roberts [7].

RNAi target sequences

Sequences of the used siRNAs are: human Efemp1-1: 5` G A U A U U G A U G A A U G U G A C A 3`; human Efemp1-2: 5` G U G U G U C A A C C A C U A U G G A 3`; murine Efemp1-1: 5` U G A G C A U C C U C A G C A A G A A 3`; murine Efemp1-2: 5` U A U A C A C C U C C A C A G A C C A 3` and control siRNA: 5` C A G U C G C G U U U G C G A C U G G 3`.

Antibodies

The following antibodies were used: Phospho-AKT (Ser473) (Cell Signaling, #4060), pan-AKT (Cell Signaling, #4691), Phospho-S6 (Ser235/236) (Cell signaling, #4856), pan-S6 (Cell signaling, #2317), SKP2 (H-435, sc-7164, Santa Cruz Biotechnology), p27$^{\text{Kip1}}$ (C-19, sc-528, Santa Cruz Biotechnology), α-tubulin (Sigma-Aldrich, T6199), HDAC1 (05-614, Millipore) and β-actin (Sigma-Aldrich, A5316).

qPCR primer sequences

muEfemp1-fwd: 5` G C A A T G C T G G T G C T T G T G A A 3`, muEfemp1-rev: 5`A C A G A G C T T G T G C G G A A G G T T 3`, huEfemp1-fwd: 5` G A G G G G A G C A G T G C G T A G A C A 3`, huEfemp1-rev: 5`T C G G C A C A T G G C A T T T G A G A C 3`, mup27-fwd: 5`G T G G A C C A A A T G C C T G A C T C 3`, mup27-rev: 5’ T C T G T T C T G T T G G C C C T T T T 3`, muCyclophilin-fwd: 5`A T G G T C A A C C C C A C C G T G T 3`, muCyclophilin-rev: 5`T T C T T G C T G T C T T T G G A A C T T T G T C 3`, huCyclophilin-fwd: 5`A T G G T C A A C C C C A C C G T G T 3`, huCyclophilin-rev: 5`T C T G C T G T C T T T G G G A C C T T G T C 3` .

SA-β-galactosidase staining

For analysis of cellular senescence, cells were washed three times with PBS (pH 7.4) and fixed for 5 minutes with 2% paraformaldehyde at room temperature. After three washing steps with PBS (pH 7.4) the cells were incubated with freshly prepaired X-Gal-staining solution (1 mg/ml X-Gal, 40mM citric acid/sodium phosphate (pH 6.0), 5mM potassium ferrocyanide, 5mM potassium ferricyanide, 150mM NaCl and 2mM $MgCl_2$) at 37°C for 6 hours.

Analysis of Microarrays

The raw microarray probe intensities are processed with standard open-source bioinformatics tools to account for technical variation and to quantitate gene expression levels. We use the method VSN [8] with standard parameters to summarize the different types of probes into probeset-level expression values, to correct for the microarray specific background intensity and to normalize the signal between different samples. Calculated expression values are log2 transformed for further analyses. Statistical significance of differential expression and (log) fold change for each probeset was assessed with the R Bioconductor package “Limma” [9]. To account for the number of tests (45.000 probesets) multiple testing correction was done by converting p-values into local false discovery rates [10]. Probesets are mapped to transcripts and genes with the current annotation provided by Affymetrix for the GeneChip Mouse

Genome 430 2.0. Clustering of differential expressed genes was performed by calculating the euclidean distance matrix between all gene expression profiles. Genes and samples are clustered according to this similarity measure and ordered in a hierarchical dendrogram.

Stable transfection

For stable transfection, linearized pCMV6-Efemp1 or pCMV6 plasmids were transfected using polyethylenimine (Sigma-Aldrich) as recently described [11]. pCMV6-Efemp1 was purchased from OriGene Technologies, Rockville, USA (MC201776). Twenty-four hours post-transfection, cells were serial diluted and cultured in medium containing 800 μg/ml Geneticin (Invitrogen). After two weeks, single clones were picked, propagated, and analyzed for Efemp1 expression. MiaPaCa2 cells stably transfected with pcDNA3 or pcDNA3-Ha-Skp2 were described [12].

RCAS virus construction and RCAS virus transduction

The RCASBP(A)-EGFP vector was recently described [4]. For generation of the RCASBP(A)-EGFP-Flag-SKP2 vector, the Flag-SKP2 expression cassette was excised from pcDNA3-Flag-SKP2 plasmid (kindly provided by Dr. Nakayama [13]) by XhoI digestion and blunt ended inserted into the PmeI site of the pEntr™ / D-Topo-EGFP vector (Invitrogen, Karlsruhe, Germany), which was modified with an EF1α-EGFP cassette. The Flag-SKP2-EF1α-EGFP cassette was transferred into the viral RCASBP(A) vector using Gateway® LR clonase™ enzyme mix (Invitrogen, Karlsruhe, Germany).

Transduction of the TvA expressing murine PPT-6554 cell line with RCASBP(A)-EGFP and RCASBP(A)-EGFP-Flag-SKP2 was done as recently described [4, 14].

References: Supplementary Material and Methods

1. Hingorani SR, Petricoin EF, Maitra A, Rajapakse V, King C, Jacobetz MA, Ross S, Conrads TP, Veenstra TD, Hitt BA, Kawaguchi Y, Johann D, Liotta LA, Crawford HC, Putt ME, Jacks T, et al. Preinvasive and invasive ductal pancreatic cancer and its early detection in the mouse. Cancer Cell. 2003; 4(6):437-450.

2. Nakhai H, Sel S, Favor J, Mendoza-Torres L, Paulsen F, Duncker GI and Schmid RM. Ptf1a is essential for the differentiation of GABAergic and glycinergic amacrine cells and horizontal cells in the mouse retina. Development. 2007; 134(6):1151-1160.

3. Olive KP, Tuveson DA, Ruhe ZC, Yin B, Willis NA, Bronson RT, Crowley D and Jacks T. Mutant p53 gain of function in two mouse models of Li-Fraumeni syndrome. Cell. 2004; 119(6):847-860.

4. Seidler B, Schmidt A, Mayr U, Nakhai H, Schmid RM, Schneider G and Saur D. A Cre-loxP-based mouse model for conditional somatic gene expression and knockdown in vivo by using avian retroviral vectors. Proc Natl Acad Sci U S A. 2008; 105(29):10137-10142.

5. Eser S, Reiff N, Messer M, Seidler B, Gottschalk K, Dobler M, Hieber M, Arbeiter A, Klein S, Kong B, Michalski CW, Schlitter AM, Esposito I, Kind AJ, Rad L, Schnieke A, et al. Selective requirement of PI3K/PDK1 signalling for Kras oncogene-driven pancreatic cell plasticity and cancer. Cancer Cell. 2013; in press.

6. Jonkers J, Meuwissen R, van der Gulden H, Peterse H, van der Valk M and Berns A. Synergistic tumor suppressor activity of BRCA2 and p53 in a conditional mouse model for breast cancer. Nat Genet. 2001; 29(4):418-425.

7. Fero ML, Rivkin M, Tasch M, Porter P, Carow CE, Firpo E, Polyak K, Tsai LH, Broudy V, Perlmutter RM, Kaushansky K and Roberts JM. A syndrome of multiorgan hyperplasia with features of gigantism, tumorigenesis, and female sterility in p27(Kip1)-deficient mice. Cell. 1996; 85(5):733-744.

8. Huber W, von Heydebreck A, Sultmann H, Poustka A and Vingron M. Variance stabilization applied to microarray data calibration and to the quantification of differential expression. Bioinformatics. 2002; 18 Suppl 1:S96-104.

9. Smyth GK, Yang YH and Speed T. Statistical issues in cDNA microarray data analysis. Methods Mol Biol. 2003; 224:111-136.

10. Benjamini Y and Hochberg Y. Controlling the False Discovery Rate: A Practical and Powerful Approach to Multiple Testing. Journal of the Royal Statistical Society. 1995; 57(1):289-300.

11. Wirth M, Fritsche P, Stojanovic N, Brandl M, Jaeckel S, Schmid RM, Saur D and Schneider G. A simple and cost-effective method to transfect small interfering RNAs into pancreatic cancer cell lines using polyethylenimine. Pancreas. 2011; 40(1):144-150.

12. Schneider G, Saur D, Siveke JT, Fritsch R, Greten FR and Schmid RM. IKKalpha controls p52/RelB at the skp2 gene promoter to regulate G1- to S-phase progression. EMBO J. 2006; 25(16):3801-3812.

13. Kamura T, Hara T, Kotoshiba S, Yada M, Ishida N, Imaki H, Hatakeyama S, Nakayama K and Nakayama KI. Degradation of p57Kip2 mediated by SCFSkp2-dependent ubiquitylation. Proc Natl Acad Sci U S A. 2003; 100(18):10231-10236.

14. von Werder A, Seidler B, Schmid RM, Schneider G and Saur D. Production of avian retroviruses and tissue-specific somatic retroviral gene transfer in vivo using the RCAS/TVA system. Nat Protoc. 2012; 7(6):1167-1183.
